# Study of the Impact of Antibiotic Combinations Used in Urinary Tract Infections on the Effectiveness of Antimicrobial Therapy

**DOI:** 10.3390/jcm15103947

**Published:** 2026-05-20

**Authors:** Jagoda Jeziurska-Pavlenko, Dagmara Fydrych, Joanna Kwiecińska-Piróg, Jana Wełna, Emilia Siemińska

**Affiliations:** Department of Microbiology, Collegium Medicum of L. Rydygier in Bydgoszcz, Nicolaus Copernicus University in Toruń, 9 M. Skłodowskiej-Curie Street, 85-094 Bydgoszcz, Poland; jagoda.jeziurska@doktorant.umk.pl (J.J.-P.); 503577@doktorant.umk.pl (D.F.); jana.przekwas@doktorant.umk.pl (J.W.);

**Keywords:** biofilm, urinary tract infections, uropathogens, ciprofloxacin, nitrofurantoin, amikacin, imipenem

## Abstract

**Background:** Biofilm-associated urinary tract infections (UTIs) pose a significant therapeutic challenge due to the increased tolerance of biofilm-embedded bacteria to antimicrobial agents and the high risk of infection recurrence. The increasing prevalence of multidrug-resistant uropathogens necessitates the evaluation of alternative therapeutic strategies, including antibiotic combination therapy. This study aimed to assess the antibiofilm activity of selected antibiotics used individually and in combination against biofilms formed by clinically relevant uropathogens. **Methods:** Biofilms of *Escherichia coli*, *Pseudomonas aeruginosa*, *Proteus mirabilis*, and *Enterococcus faecalis* isolated from patients with UTIs were developed on polystyrene microtiter plates and exposed to ciprofloxacin, nitrofurantoin, amikacin, and imipenem applied as monotherapy and in combinations. Biofilm biomass reduction was quantified spectrophotometrically using crystal violet staining and expressed as a percentage relative to untreated controls. **Results:** Antibiotic monotherapy produced moderate reductions in biofilm biomass, with efficacy dependent on bacterial species and antibiotic concentration. In contrast, antibiotic combinations demonstrated enhanced antibiofilm activity. The ciprofloxacin–nitrofurantoin combination showed increased biofilm biomass reduction compared with monotherapy against *P. aeruginosa* and *E. coli*. The imipenem–amikacin combination reduced *P. mirabilis* biofilm biomass by over 80%. **Conclusions:** These findings suggest that rationally selected antibiotic combinations may represent a more effective strategy than monotherapy for controlling biofilm-associated UTIs.

## 1. Introduction

Urinary tract infections (UTIs) are among the most common bacterial infections and remain a major clinical problem due to their high recurrence rate and increasing antimicrobial resistance [1,2,3,4]. Biofilm formation plays a central role in the persistence of chronic and catheter-associated urinary tract infections (CAUTIs), contributing to reduced susceptibility to antimicrobial therapy and increased tolerance to host immune responses [5,6,7]. Within biofilms, bacterial cells exhibit altered metabolic activity, restricted antimicrobial penetration, and adaptive stress responses, which may substantially reduce the efficacy of conventional antibiotic monotherapy [8,9].

The most common uropathogens associated with biofilm-related UTIs include *Escherichia coli*, *Proteus mirabilis*, *Pseudomonas aeruginosa*, and *Enterococcus faecalis* [5,10]. Among these, *E. coli* remains the dominant etiological agent of uncomplicated UTIs, whereas *P. mirabilis* and *P. aeruginosa* are frequently associated with catheter-related and chronic infections due to their strong biofilm-forming capacity [4,5,10]. The increasing prevalence of multidrug-resistant (MDR) strains further complicates treatment and highlights the need for alternative therapeutic strategies capable of improving antimicrobial activity under biofilm-associated conditions [2,11].

Combination antibiotic therapy has been proposed as one possible approach to improve antibiofilm activity and potentially overcome some limitations of monotherapy [12]. However, antibiotic interactions observed in planktonic susceptibility testing do not necessarily reflect behavior under biofilm conditions, where altered bacterial physiology may substantially modify antimicrobial responses [8,13]. Consequently, evaluation of antibiotic combinations in biofilm-associated models may provide insight into interaction patterns that are not apparent in conventional susceptibility systems.

The present study evaluated the effects of selected antibiotics used individually and in combination against biofilms formed by clinically relevant uropathogens. Ciprofloxacin, nitrofurantoin, amikacin, and imipenem were selected because they represent antimicrobial agents commonly used in the management of urinary tract infections or multidrug-resistant Gram-negative infections. Some tested combinations were included primarily as exploratory in vitro biofilm models intended to investigate how biofilm-associated bacterial physiology may influence biomass-related responses to combined antimicrobial exposure rather than to simulate guideline-preferred therapeutic regimens. In particular, nitrofurantoin against *P. aeruginosa*, ciprofloxacin against *E. faecalis*, and imipenem against *P. mirabilis* should be interpreted cautiously from a translational perspective.

Four drug combinations were used in the study:

(1) Ciprofloxacin x amikacin: *E.coli*, *P. aeruginosa*

The combination of these antibiotics appears particularly promising, especially in the treatment of infections associated with colonization of medical devices. Ciprofloxacin is characterized by high bioavailability and excellent penetration into deeper tissues, including the deepest layers of bacterial biofilm. Moreover, the primary action of ciprofloxacin is based on the inhibition of DNA gyrase. This prevents double-stranded DNA from closing, leading to exonucleolytic degradation. This inhibits the synthesis of proteins, enzymes, and bacterial toxins, leading to bacterial cell death [14]. Amikacin also inhibits bacterial protein synthesis. It exerts its effect by irreversibly binding to the bacterial 30S ribosomal subunit, leading to erroneous mRNA reading [15]. Thanks to this, amikacin is the most widely acting aminoglycoside and, according to the latest guidelines, is recommended for the treatment of acute *P. aeruginosa* infections. It is also the least nephrotoxic antibiotic [16].

(2) Ciprofloxacin x nitrofurantoin: *E. faecalis*

This combination is not used in clinical practice due to reported antagonistic interactions under standard susceptibility testing conditions. However, given the distinct physiology of biofilm-associated bacteria, evaluating this combination in an in vitro biofilm model may provide insight into potential interactions that are not apparent in planktonic systems. Although ciprofloxacin may retain limited clinical utility in selected uncomplicated urinary tract infections caused by *Enterococcus* spp., it is not considered a preferred agent for chronic biofilm-associated enterococcal infections. In the present study, ciprofloxacin was included primarily to assess how antibiotic interaction behavior may change under biofilm-associated conditions compared with conventional planktonic susceptibility models. Thus, the evaluated combination should be interpreted as an exploratory in vitro biofilm model rather than a simulation of guideline-preferred therapeutic management.

Nitrofurantoin is a promising antibiotic with potent bactericidal properties. Due to its intracellular reduction in bacterial cells to reactive metabolites that damage DNA, RNA, proteins, and cell membranes, it leads to rapid bacterial cell death [17]. Moreover, compared to ciprofloxacin, resistance to nitrofurantoin is significantly less common [18]. Thanks to this combination, the antibiotic complex has the potential to penetrate bacterial biofilms, which would prevent resistance when using ciprofloxacin alone. It is worth mentioning collateral resistance, the acquisition of which can cause mutations in bacterial cells. The resulting acquired genes can enhance or even inhibit the action of a previously ineffective antibiotic [19].

(3) Imipenem x amikacin: *P. mirabilis*

Imipenem, a member of the carbapenem group, is a last-resort antibiotic among routinely used antibiotics. These drugs are highly taxing to the liver and kidneys, causing more side effects than other classes of chemotherapeutic agents. They also exhibit a strong antimicrobial effect by inhibiting cell wall synthesis and inactivating penicillin-binding proteins, ultimately leading to bacterial cell lysis and death [15]. Carbapenems are an effective therapeutic solution, especially when strains are sensitive to them. Unfortunately, in recent years, microbial resistance to even this group of drugs has been increasing and spreading. Combination therapy with carbapenems and aminoglycosides is used for multidrug-resistant infections in immunocompromised patients who have repeatedly received broad-spectrum antibiotics. This therapy can reduce antibiotic doses and limit the development of resistance [20]. Although carbapenems are used in the management of severe multidrug-resistant Gram-negative infections, the inclusion of imipenem in the present *P. mirabilis* biofilm model should be interpreted cautiously. Members of the Morganellaceae family may demonstrate variable susceptibility to carbapenems, and imipenem does not necessarily represent a preferred first-line therapeutic option for Proteus-associated urinary tract infections. Therefore, this combination was included primarily as an exploratory in vitro model to evaluate how biofilm-associated bacterial physiology may influence biomass-related responses to combined antimicrobial exposure rather than to simulate a guideline-preferred therapeutic regimen.

(4) Amikacin x nitrofurantoin: *E. coli*

In vitro studies confirm the increased reduction effects of these two antibiotics in combination compared to monotherapy. Both are used in the routine treatment and prevention of urinary tract infections. These drugs may potentiate each other’s effects. Nitrofurantoin generates reactive oxygen species in bacterial cells, which in turn facilitates the penetration of aminoglycosides. Once inside the bacterial cell, their common targets are the ribosomes and disrupt the bacterial cell’s genetic expression [21].

The present study was designed to explore potential interactions between selected antibiotics under biofilm conditions. Unlike planktonic susceptibility testing, biofilm-associated bacteria exhibit phenotypes driven by reduced growth rate, extracellular matrix-mediated diffusion barriers, altered metabolic states, and tolerance mechanisms that may modify antibiotic interactions. As a result, antibiotic combinations that appear additive or indifferent in planktonic systems may behave differently in biofilms. The combinations tested here were selected to determine whether pairing agents with distinct mechanisms of action could enhance biofilm biomass reduction compared with monotherapy.

## 2. Materials and Methods

### 2.1. Materials

**Figure 1:**
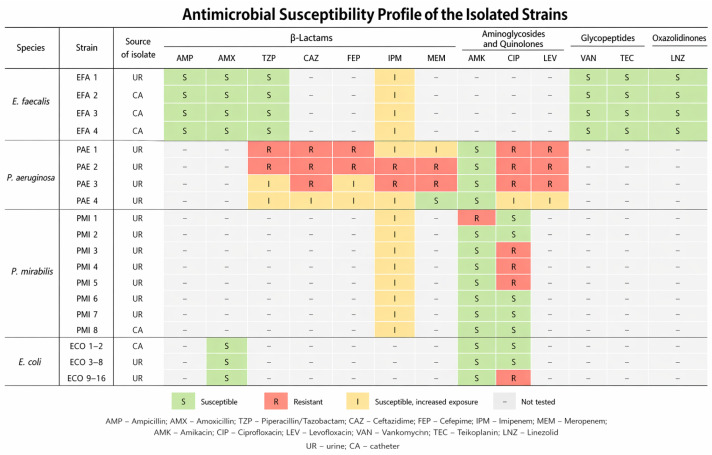
The antimicrobial susceptibility profile of the isolated strains.

The study included 32 strains: 4 strains of *P. aeruginosa*, 4 strains of *E. faecalis*, 16 strains of *E. coli* and 8 strains of *P. mirabilis*. The antimicrobial susceptibility profile of the isolated strains is presented in Figure 1. The clinical strains used for the study are part of the collection of the Department of Microbiology, Ludwik Rydygier Medical College in Bydgoszcz, Nicolaus Copernicus University in Toruń. The study included data regarding the results of antibiotic susceptibility testing, which was downloaded from the Promic IT system (Mori^®^, Marcin Bogucki, Ostaszewo, Poland). Antibiotic susceptibility was determined as part of microbiological diagnostics performed by staff at the University Hospital No. 1 named after Dr. Antoni Jurasz in Bydgoszcz using an automated method (Phoenix M50, Becton Dickinson, Franklin Lakes, NJ, USA).

### 2.2. Methods

The tested strains were stored at −70 °C until analysis. Each strain was tested in three independent biological experiments to ensure reproducibility. Prior to experimentation, strains were subcultured twice on Columbia sheep blood agar and incubated for 24 h at 37 °C. A bacterial suspension equivalent to 0.5 McFarland standard was prepared in sterile saline using colonies obtained from fresh overnight cultures and verified by densitometry.

Biofilm formation and antibiotic exposure were performed in sterile 96-well polystyrene microplates. Each well contained 100 μL Mueller–Hinton broth (MHB), bacterial suspension, and the tested antibiotic solution. Ciprofloxacin, nitrofurantoin, amikacin, and imipenem (Sigma-Aldrich, St. Louis, MO, USA) were prepared in MHB and applied using serial two-fold (1:2) dilutions. Antibiotic concentration ranges included sub-MIC, near-MIC, and supra-MIC exposures to evaluate biomass-associated responses under different antimicrobial conditions. Plates were incubated for 24 h at 37 °C.

After incubation, the contents of the wells were removed, and the wells were washed three times with sterile distilled water to remove non-adherent cells. Plates were then dried at 37 °C for 20 min. Biofilm biomass was quantified using 0.1% crystal violet (CV; Avantor, Gliwice, Poland), with 200 μL added to each well. Staining was performed for 20 min under agitation at 400 rpm to facilitate dye binding. Excess unbound dye was removed, and the wells were washed with distilled water until colorless washings were obtained. Plates were dried again at 37 °C for 20 min. Subsequently, 200 μL of 96% ethanol (ChemPur, Piekary Śląskie, Poland) was added to each well to solubilize the bound crystal violet, followed by agitation for 5 min. Absorbance was measured at 570 nm using a Synergy HT microplate spectrophotometer (BioTek, Winooski, VT, USA).

Positive control wells contained bacterial suspension with MHB, whereas negative control wells contained sterile MHB only. Absorbance values were corrected by subtraction of blank values obtained from negative control wells. The percentage biofilm biomass reduction was calculated relative to untreated control biofilms.

**Figure 2:**
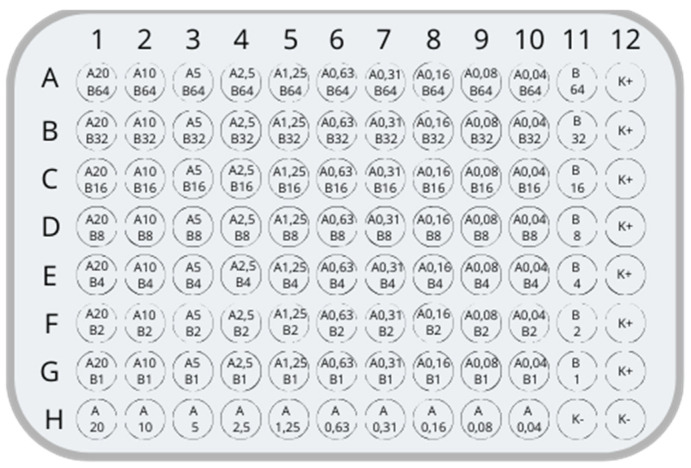
The system of concentrations acting on microorganisms in the study. (K+): positive control, (K−): negative control, A: drug A concentration in μg/mL, B: drug B concentration in μg/mL. For example, A20: drug A with a concentration of 20 μg/mL. Prepared by Canva (Sydney, Australia).

For each tested strain, multiple antibiotic concentration conditions were evaluated. The highest percentage biofilm biomass reduction observed for a given treatment condition was selected for further comparative statistical analysis. Mean values and standard deviations presented in the Results section were subsequently calculated from these maximal reduction values obtained for individual strains within each bacterial species group (Figure 2, Table 1).

### 2.3. Calculation of the Degree of Biofilm Reduction

To obtain the normalized values of the absorbance change in the biofilm mass (AZ) formed in the wells after incubation, according to the following formula:

$$AZ=\;\frac{\left(AK+)-(AB\right)}{(AK+)\;\times \;100\%}$$

(AK+): Absorbance of the positive control;

(AB): Absorbance of the biofilm formed in the plate is well-drawn.

For each tested strain, multiple antibiotic concentration conditions were evaluated. The highest percentage biofilm biomass reduction observed for a given strain under antibiotic concentration was selected for further comparative statistical analysis. Mean values and standard deviations presented in the Results section were subsequently calculated from these maximal reduction values obtained for individual strains within each bacterial species group.

## 3. Results

Results are presented as mean percentage biofilm reduction ± standard deviation (SD), calculated at the level of independent strains. The efficacy of monotherapy was compared with the corresponding antibiotic combinations. Statistical significance between monotherapy and combination therapy was evaluated using a two-tailed Welch’s *t*-test, which was selected to account for unequal variances between groups; *p* < 0.05 was considered statistically significant. These results are summarized in Table 2.

### 3.1. Effects of Antibiotic Treatment Against E. faecalis (n = 4)

Ciprofloxacin monotherapy produced a mean biofilm reduction of 28.70 ± 10.93%, whereas the ciprofloxacin + nitrofurantoin combination achieved 19.33 ± 8.49%. Combination therapy showed lower activity than monotherapy, suggesting an antagonistic effect. However, the difference was not statistically significant (*p* = 0.22).

### 3.2. Effects of Antibiotic Treatment Against P. aeruginosa (n = 4)

Ciprofloxacin monotherapy resulted in a mean biofilm reduction of 29.46 ± 11.17%, whereas the ciprofloxacin + nitrofurantoin combination increased the reduction to 43.75 ± 9.52%. A trend toward greater efficacy of combination therapy was observed, although the difference did not reach statistical significance (*p* = 0.10).

### 3.3. Effects of Antibiotic Treatment Against E. coli (n = 16)

For the ciprofloxacin + amikacin combination, mean biofilm reduction was 43.41 ± 7.17%, significantly higher than ciprofloxacin monotherapy (39.06 ± 4.40%; *p* = 0.041), showing significantly greater biofilm biomass reduction than ciprofloxacin alone.

For the nitrofurantoin + amikacin combination, mean biofilm reduction was 35.96 ± 12.92%, compared with 32.41 ± 10.38% for the best-performing monotherapy (amikacin), but this difference was not statistically significant (*p* = 0.39).

### 3.4. Effects of Antibiotic Treatment Against P. mirabilis (n = 8)

Imipenem monotherapy showed a mean biofilm reduction of 58.78 ± 6.52%, while the imipenem + amikacin combination increased the reduction to 62.49 ± 9.68%. This increase was modest and did not reach statistical significance (*p* = 0.37).

## 4. Discussion

This study assessed the activity of ciprofloxacin, nitrofurantoin, amikacin, and imipenem, used alone and in combination, against biofilms of *E. faecalis*, *P. aeruginosa*, *E. coli*, and *P. mirabilis* isolated from urine. Because biofilm-associated bacteria differ fundamentally from planktonic cells in growth state, antibiotic penetration, and tolerance mechanisms, antibiotic interactions observed in biofilms may differ substantially from those predicted from conventional susceptibility testing. This makes evaluation of combination therapy under biofilm conditions particularly relevant.

Ciprofloxacin reduced *E. faecalis* biofilm, with lower efficacy observed at subinhibitory concentrations and increasing activity at higher exposure. The limited activity of fluoroquinolones against enterococci may reflect target mutations, efflux-mediated adaptive responses, and intrinsic tolerance mechanisms [16,22]. Restricted penetration through the extracellular polymeric matrix and the presence of metabolically inactive persister-like subpopulations may further reduce monotherapy performance in mature biofilms. These observations are consistent with previous reports showing limited antibiofilm activity of ciprofloxacin despite transcriptional changes in metabolic pathways [23,24]. The ciprofloxacin–nitrofurantoin combination evaluated against E. faecalis should also be interpreted cautiously from a translational perspective. Because fluoroquinolones are not considered preferred agents for chronic biofilm-associated enterococcal urinary tract infections, this experimental model was intended primarily to investigate potential interaction behavior under biofilm conditions rather than to reflect standard therapeutic practice.

Ciprofloxacin demonstrated moderate activity against *P. aeruginosa* and *E. coli* biofilms, consistent with prior reports linking activity to *gyrA/parC* status and concentration-dependent changes in biofilm physiology [12,16,25,26]. However, monotherapy responses remained incomplete, supporting the concept that biofilm tolerance mechanisms—including matrix-dependent diffusion limitation, altered metabolic states, persister-cell behavior, and inducible efflux systems—may reduce susceptibility under biofilm conditions.

Nitrofurantoin showed variable activity across species, consistent with its multitarget mechanism involving reactive intermediates [17]. Notably, in *E. faecalis*, greater activity was observed at lower concentrations, and no simple dose–response relationship was apparent. This non-linear pattern may reflect paradoxical concentration-dependent adaptation under biofilm conditions, potentially involving stress signaling or altered c-di-GMP-mediated regulation [19,21]. Such behavior may also help explain why the ciprofloxacin–nitrofurantoin combination performed worse than ciprofloxacin alone in *E. faecalis*, suggesting a possible antagonistic or adaptive interaction rather than a uniformly beneficial combination effect.

In contrast, ciprofloxacin plus nitrofurantoin showed a numerical increase in biofilm reduction in P. aeruginosa, although this effect did not reach statistical significance. This suggests a favorable but non-universal interaction, potentially related to complementary effects on oxidative stress or DNA damage pathways [27,28]. Importantly, the ciprofloxacin–nitrofurantoin combination against *P. aeruginosa* should be interpreted exclusively within the context of an exploratory in vitro biofilm model. Because nitrofurantoin lacks established clinical utility against P. aeruginosa, the present findings are not intended to support therapeutic application of this combination. Instead, the model was used to explore whether biofilm-associated bacterial physiology may modify interaction patterns observed under conventional planktonic conditions.

For *E. coli*, ciprofloxacin plus amikacin was the only combination that demonstrated statistically significant superiority over monotherapy, supporting a species- and combination-dependent benefit of dual therapy. By contrast, the amikacin–nitrofurantoin combination did not show significant superiority over the best monotherapy despite numerical improvement, indicating that improved activity was not universal across combinations. These findings are consistent with reports that combination effects may be context-dependent and not necessarily associated with resistance suppression [29,30].

The imipenem–amikacin combination showed increased activity against *P. mirabilis* biofilms relative to imipenem alone, although the difference was not statistically significant. This suggests a modest increase in activity rather than a clearly superior combination effect. Given the strong biofilm-forming capacity of *P. mirabilis* and its relevance in catheter-associated infections, this finding warrants further mechanistic investigation [29,30]. Importantly, the imipenem–amikacin combination against *P. mirabilis* should not be interpreted as evidence supporting a preferred therapeutic strategy for Proteus-associated urinary tract infections. The model was included primarily to explore potential biofilm-associated interaction patterns under controlled in vitro conditions. Because antimicrobial susceptibility and clinical utility of carbapenems against Morganellaceae may vary, the translational significance of these observations remains limited and requires cautious interpretation.

Among the tested combinations, only ciprofloxacin plus amikacin against *E. coli* demonstrated a statistically significant advantage over monotherapy. For *P. aeruginosa*, a non-significant increase in activity was observed without statistical confirmation, whereas for *E. faecalis*, the ciprofloxacin–nitrofurantoin combination showed an antagonistic trend. For *P. mirabilis* and nitrofurantoin plus amikacin against *E. coli*, no significant superiority over monotherapy was found. Together, these findings indicate that the efficacy of combination therapy is species- and drug-dependent rather than universal, arguing against the assumption that antibiotic combinations consistently outperform single-agent therapy in biofilm-associated infection models.

The tested concentration ranges were designed to include sub-MIC, near-MIC, and higher exposures to capture potential pharmacodynamic interactions, including effects occurring only under subinhibitory conditions. In this context, the lowest imipenem concentrations were included to explore biofilm-modulatory effects rather than simulate therapeutic exposure. Translational interpretation should therefore rely primarily on effects observed within clinically relevant concentration ranges, particularly for agents such as nitrofurantoin that achieve high urinary but limited tissue concentrations.

Several limitations should be acknowledged. First, the crystal violet assay measures total biofilm biomass rather than bacterial viability and therefore does not demonstrate killing, eradication, resistance suppression, or clinical treatment efficacy. Conclusions should accordingly be limited to biomass-associated effects. Second, the in vitro model does not fully reproduce urinary tract conditions such as urine flow, pH variation, or host factors. Finally, the mechanistic explanations proposed here remain hypothetical, as persister dynamics, efflux regulation, and penetration were not directly measured.

Despite these limitations, the data provide preliminary evidence that selected antibiotic combinations may improve biofilm biomass reduction in a species-dependent manner and support further investigation using viability-based assays, mechanistic studies, and translational infection models.

## 5. Conclusions

- Antibiotic monotherapy showed limited activity against mature biofilms of *E. faecalis*, *P. aeruginosa*, *E. coli*, and *P. mirabilis* isolated from urinary tract infections.
- Combination therapy was not universally superior to monotherapy; rather, its effect was species- and drug-dependent.
- Among the tested combinations, only ciprofloxacin plus amikacin against *E. coli* demonstrated statistically significant superiority over monotherapy, whereas other combinations showed either non-significant trends, no difference, or lower activity than monotherapy.
- The combinations of imipenem plus amikacin against *P. mirabilis* and ciprofloxacin plus nitrofurantoin against *P. aeruginosa* showed numerical increases in activity that warrant further investigation, but should not be interpreted as confirmed superior therapeutic strategies.
- These results provide preliminary evidence that selected antibiotic combinations warrant further evaluation in clinical models of biofilm-associated urinary tract infections.

## Figures and Tables

**Table 1:** Scheme of drugs that act on bacterial biofilms of individual microbial species.

Microorganism	Antibiotic A	Antibiotic B
*Enterococcus faecalis*	Ciprofloxacin	Nitrofurantoin
*Pseudomonas aeruginosa*	Ciprofloxacin	Nitrofurantoin
*Escherichia coli* (1)	Ciprofloxacin	Amikacin
*Escherichia coli* (2)	Amikacin	Nitrofurantoin
*Proteus mirabilis*	Imipenem	Amikacin

**Table 2:** Statistical comparison of mean biofilm reduction produced by monotherapy versus combination therapy.

Species	Antibiotic (Monotherapy/ Combination)	Mean Biofilm Reduction (%)	SD	*p*-Value	Interpretation
*E. faecalis*	Ciprofloxacin	28.70	10.93	—	Reference monotherapy
*E. faecalis*	Ciprofloxacin + Nitrofurantoin	19.33	8.49	0.22	Antagonistic trend (NS)
*P. aeruginosa*	Ciprofloxacin	29.46	11.17	—	Reference monotherapy
*P. aeruginosa*	Ciprofloxacin + Nitrofurantoin	43.75	9.52	0.10	No significant difference (NS)
*E. coli*	Ciprofloxacin	39.06	4.40	—	Reference monotherapy
*E. coli*	Ciprofloxacin + Amikacin	43.41	7.17	0.041	Significant superiority
*E. coli*	Amikacin	32.41	10.38	—	Reference monotherapy
*E. coli*	Amikacin + Nitrofurantoin	35.96	12.92	0.39	No significant difference
*P. mirabilis*	Imipenem	58.78	6.52	—	Reference monotherapy
*P. mirabilis*	Imipenem + Amikacin	62.49	9.68	0.37	No significant difference

## Data Availability

The original contributions presented in this study are included in the article. Further inquiries can be directed to the corresponding authors.
